# Oxidative Protein-Folding Systems in Plant Cells

**DOI:** 10.1155/2013/585431

**Published:** 2013-09-25

**Authors:** Yayoi Onda

**Affiliations:** Department of Food and Applied Life Sciences, Yamagata University, Yamagata 997-8555, Japan

## Abstract

Plants are unique among eukaryotes in having evolved organelles: the protein storage vacuole, protein body, and chloroplast. Disulfide transfer pathways that function in the endoplasmic reticulum (ER) and chloroplasts of plants play critical roles in the development of protein storage organelles and the biogenesis of chloroplasts, respectively. Disulfide bond formation requires the cooperative function of disulfide-generating enzymes (e.g., ER oxidoreductase 1), which generate disulfide bonds de novo, and disulfide carrier proteins (e.g., protein disulfide isomerase), which transfer disulfides to substrates by means of thiol-disulfide exchange reactions. Selective molecular communication between disulfide-generating enzymes and disulfide carrier proteins, which reflects the molecular and structural diversity of disulfide carrier proteins, is key to the efficient transfer of disulfides to specific sets of substrates. This review focuses on recent advances in our understanding of the mechanisms and functions of the various disulfide transfer pathways involved in oxidative protein folding in the ER, chloroplasts, and mitochondria of plants.

## 1. Introduction

**Figure 1:**
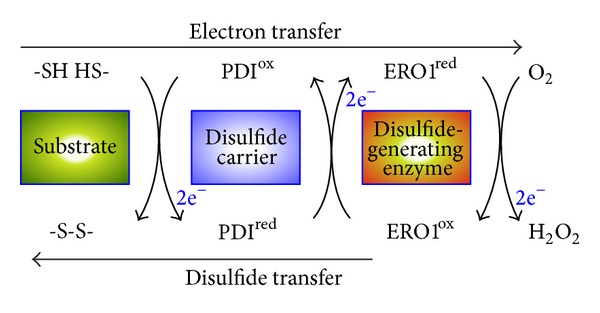
Disulfide relay from ERO1 to PDI in the ER. Disulfide bond formation involves electron transfer from the substrate to a disulfide carrier protein (e.g., PDI) and then to a de novo disulfide-generating enzyme (e.g., ERO1). PDI directly transfers a disulfide to two Cys residues of a substrate protein by means of a thiol-disulfide exchange reaction. The reduced form of PDI is reoxidized by ERO1, which relays the oxidizing power from molecular oxygen, via the FAD cofactor, to the reduced form of PDI.

The endoplasmic reticulum (ER) is the first organelle in the secretory pathway, and this dynamic and highly specialized organelle contains enzymes and chaperones that mediate the folding and assembly of newly synthesized proteins [1]. A key step in oxidative protein folding is the formation of disulfide bonds, which covalently link the side chains of pairs of Cys residues, impart thermodynamic and mechanical stability to proteins, and control protein folding and activity [2]. The introduction of disulfide bonds into polypeptides requires the cooperative function of a pair of enzymes, a de novo disulfide-generating enzyme (e.g., ER oxidoreductase 1 [ERO1]) and a disulfide carrier protein (e.g., protein disulfide isomerase [PDI]) [3]. PDIs, which are ubiquitous thiol-disulfide oxidoreductases in all eukaryotic cells, directly donate disulfides to substrate proteins by means of thiol-disulfide exchange reactions. The oxidized form of PDI is regenerated by ERO1, which relays the oxidizing power from molecular oxygen to the reduced form of PDI (Figure 1). The genomes of higher plants, including *Arabidopsis thaliana*, *Glycine max *(soybean), *Oryza sativa* (rice), and* Zea mays *(maize), encode approximately 10 to 20 members of the PDI family, which show wide variation in the organization of their thioredoxin (TRX)-fold domains [4, 5]. Although the network of enzymes involved in disulfide bond formation has not yet been fully elucidated, emerging evidence suggests that the molecular and structural diversity of the PDIs and the specific combinations of disulfide-generating enzymes and PDIs are key to determining the functions of these enzymes and their substrate specificity.

Plant cells have two distinct types of vacuoles: the prototypical lytic vacuole (LV), which has high hydrolytic activity and shares functions with the yeast vacuole and mammalian lysosome, and the protein storage vacuole (PSV), a plant-specific organelle that is specialized in accumulating reserve proteins and is prevalent in seeds [6]. The developing endosperm cells of seeds actively synthesize large amounts of storage proteins that acquire disulfide bonds in the ER. Among the physicochemically and structurally diverse disulfide-rich seed storage proteins, soluble storage proteins are transported through the endomembrane system from the ER to PSVs, where they accumulate [7]. Insoluble proteins, however, form large oligomeric accretions within the ER and are deposited in ER-derived protein bodies (PBs), which bud and disconnect from the ER but remain surrounded by ER membranes [8]. The development of the PSVs and PBs enables massive, structurally stable accumulations of storage proteins, which serve as nutritional reserves of nitrogen, sulfur, and carbon for the germinating seedlings (reviewed in [9, 10]). The redox state of storage proteins containing disulfide groups dramatically changes during seed development, maturation, and germination [11]; seed storage proteins are synthesized in reduced form on the rough ER and are converted to disulfide-bonded forms during maturation; then they are converted back to the reduced form to facilitate rapid mobilization during germination. The *h*-type TRX functions in the reduction of seed storage proteins in the endosperm, increasing their proteolytic susceptibility and making nitrogen and sulfur available during germination [12]. In contrast to the TRX-dependent system that acts mainly in the reduction of disulfides, the disulfide transfer systems involving ERO1 and PDIs function primarily to form the correct patterns of disulfide bonds, thereby facilitating the stable accumulation of seed storage proteins, preventing their denaturation, and decreasing their susceptibility to proteolysis.

In chloroplasts, the redox state of disulfide bonds in redox-regulated proteins is linked to the control of metabolic pathways and gene expression [11, 13, 14]. The most-studied redox regulatory system is the ferredoxin-TRX system that reversibly reduces and oxidizes thiol groups; reducing equivalents are transferred from photosystem I (PSI) to ferredoxin and then to TRX via ferredoxin-dependent TRX reductase [11, 13]. Inside chloroplasts, the thylakoid membranes, which enclose the lumen that originated as the bacterial periplasm, perform the primary events of oxygenic photosynthesis. Under strong illumination, however, photosystem II (PSII) in the thylakoid membranes is subjected to photodamage. To maintain photosynthetic activity, PSII requires the rapid reassembly of a thylakoid membrane protein complex composed of dozens of proteins [15], which is dependent on disulfide bond formation catalyzed by enzyme catalysts, including a zinc finger protein LQY1 and a vitamin K epoxide reductase (VKOR) homolog.

**Figure 2:**
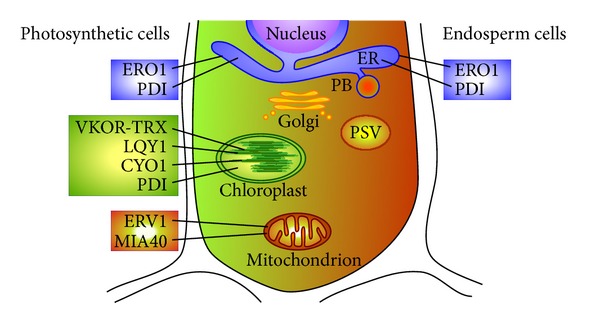
Overview of the sites of oxidative protein folding in photosynthetic and endosperm cells of plants. Disulfide-generating enzymes and disulfide carrier proteins characterized to date and their subcellular localizations are shown in photosynthetic (left) and endosperm (right) cells of plants. ER, endoplasmic reticulum; PB, protein body; PSV, protein storage vacuole.

Emerging evidence has shown that disulfide transfer pathways that function in the ER and chloroplasts of plants play critical roles in the development of PSVs and PBs and the biogenesis of chloroplasts, respectively. This review focuses on recent advances in our understanding of the mechanisms and functions of the various disulfide transfer pathways involved in oxidative protein folding in the ER and chloroplasts of plants (Figure 2). The disulfide transfer pathways in plant mitochondria, which involve the disulfide-generating enzyme ERV1 and the disulfide carrier protein MIA40 (Figure 2), are also discussed in comparison to the respective pathways in yeast and mammalian cells.

## 2. Disulfide Bond Formation in the Plant ER

### 2.1. Seed Storage Proteins

Seed storage proteins are conventionally classified on the basis of their solubility in water (e.g., albumins), saline (e.g., *α*-globulin), alcohol (e.g., prolamins), and acidic or basic solutions (e.g., glutelins) [16]. The globulins, the most widely distributed group of storage proteins in both dicots and monocots, are divided into two subgroups on the basis of their sedimentation coefficients: the vicilin-like 7S globulins and the legumin-like 11S globulins. Rice glutelins (60% of total seed protein) [17] and soybean glycinins (40% of total seed protein) [18], both of which are 11S globulin homologs, are synthesized from their precursors, proglutelins and preproglycinins, respectively; the precursors are processed at a conserved Asp-Gly site by an asparaginyl endopeptidase [19, 20] to produce acidic and basic polypeptide subunits, which remain linked by disulfide bonds [21].

**Figure 3:**
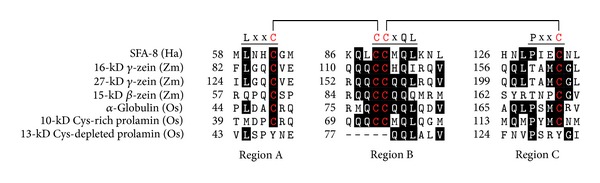
Conserved Cys residues in members of the prolamin superfamily. Amino acid sequences in regions A, B, and C of *Zea mays* (Zm) and *Oryza sativa* (Os) storage proteins are compared to *Helianthus annuus* (Ha) SFA-8. Amino acid sequences of SFA-8 (CAA40015), 16-kD *γ*-zein (NP_001105337), 27-kD *γ*-zein (NP_001105354), 15-kD *β*-zein (NP_001106004), *α*-globulin (BAA09308), and Cys-rich 10-kD prolamin (crP10; NP_001051380) were aligned by means of CLUSTALW. For comparison, the corresponding sequence of Cys-depleted 13-kD prolamin (cpP13; NP_001055213) is shown. Two disulfide bonds in SFA-8, Cys62–Cys89 and Cys90–Cys132, are indicated with solid lines. Red-on-black letters indicate Cys residues; white-on-black letters indicate amino acid residues conserved in at least four of the sequences analyzed.

The prolamin family, which includes maize prolamins (referred to as zeins) and rice prolamins, is encoded by multiple genes and is composed of Cys-rich and Cys-poor members [16, 22]. Rice prolamins (20% of total seed protein) [17] are clustered into three subgroups: 13-kD prolamins containing 0–8 Cys residues, 10-kD prolamins containing 9–11 Cys residues, and 16-kD prolamin containing 13 Cys residues [22]. Maize prolamins include four structurally distinct types of proteins: *α*-, *β*-, *γ*-, and *δ*-zeins. The *β*-, *γ*-, and *δ*-zeins are rich in Cys residues, whereas the *α*-zeins are Cys poor [16]. Prolamins and 2S albumins contain conserved motifs in three separate regions [16]: LxxC in region A, CCxQL in region B, and PxxC in region C. Cys-rich prolamins of rice and the *β*- and *γ*-zeins of maize, but not the *δ*-zeins, contain the conserved Cys residues found in regions A, B, and C (Figure 3) [16, 22]. Structural analysis shows that the sunflower (*Helianthus annuus*) 2S albumin SFA-8 forms a disulfide bond between regions A and B (between Cys62 of LxxC^62^ and Cys89 of C^89^CxQL) and one between regions B and C (between Cys90 of CC^90^xQL and Cys132 of PxxC^132^) [23]. These conserved Cys residues are also found in other members of the prolamin family, including a 10-kD Cys-rich prolamin from rice and a 16-kD *γ*-zein from maize (Figure 3) [22].

### 2.2. Protein Storage Organelles

To enable the stable accumulation of massive amounts of seed storage proteins and prevent their degradation, plants have evolved specialized membrane-bound storage organelles: PSVs that contain matrix and crystalloid components and PBs derived from the ER [10]. The multisubunit 7S and 11S globulins are synthesized in precursor form and then transported from the lumen of the rough ER to PSVs, where these proteins undergo proteolytic cleavage and assemble to form dense accretions [24]. In contrast, prolamins generally form PBs directly within the lumen of the rough ER. PBs in maize and rice remain in the ER lumen, whereas those in wheat and barley are transported from the ER to PSVs by an autophagic process [25].

Disulfide bonds play a critical role in the accumulation of seed storage proteins in PSVs and PBs [26, 27]. In the endosperm cells of rice seeds, proglutelins acquire intramolecular disulfide bonds in the ER before they are targeted, via the Golgi, to a PSV (designated type-II PB [PB-II]; a crystalloid structure with a diameter of 2–4 *μ*m). They are processed into acidic (37–39 kD) and basic (22–23 kD) subunits by a vacuolar processing enzyme (VPE) and accumulate as higher-order complexes held together by intermolecular disulfide bonds and hydrophobic interactions in the crystalloids of PSV [28–31]. The formation of intramolecular disulfide bonds is also required for the sorting of *α*-globulins to the matrix of PSV [27]. The 10-kD Cys-rich prolamins (Figure 3), however, directly accumulate within the ER of rice endosperm cells to form the center core region of an ER-derived PB (designated type-I PB [PB-I]; a spherical structure with a diameter of 1–2 *μ*m) at early stages of PB development, before the 13-kD Cys-depleted prolamins (Figure 3) are localized to the outer layer of ER-derived PB [32, 33]. The assembly of prolamins into the ER-derived PB is stabilized by the formation of intermolecular disulfide bonds [34, 35]. In the endosperm cells of maize seeds, however, the *β*- and *γ*-zeins form spherical accretions (with a diameter of 0.2 *μ*m) at early stages of PB development. Subsequently, the *α*-zeins penetrate the network of the *β*- and *γ*-zeins and fill the central region of the PB (which has a diameter of 1–2 *μ*m) [36, 37].

### 2.3. Roles of PDIs in the Development of Protein Storage Organelles: Different Subcellular Localizations and Substrate Specificities

PDIs are multifunctional enzymes that catalyze a wide range of thiol-disulfide exchange reactions, including oxidation, reduction, and isomerization reactions, and also display chaperone activity [38]. PDIs are multidomain proteins, which have at least one redox-active TRX domain, designated domain a. The oxidized form of the redox-active CxxC motif in the a domain transfers a disulfide to a substrate and is converted to the reduced form. Members of the PDI family vary widely in the number and arrangement of their a domains and of another type of TRX domain, designated domain b, which has a similar TRX-like structure but lacks the redox-active motif. For example, Pdi1p, one of the five PDIs encoded by the *Saccharomyces cerevisiae* genome (and the only one that is essential for viability [39]), has been described as a U-shaped molecule composed of four TRX-fold domains (a-b-b′-a′) and an acidic C-terminal tail; two redox-active CGHC motifs are in the N-terminal and C-terminal TRX domains (a and a′, resp.), and face each other on either side of the U shape [40]. The subsequently determined crystal structure indicates that the shape the yeast Pdi1p adopts is more that of a boat, with the b and b′ domains at the bottom and the a and a′ domains at the bow and stern [41]. The flexible arms composed of the a and a′ domains are connected to the relatively rigid core composed of the b and b′ domains, resulting in a highly flexible structure [41]. The primary substrate-binding sites of yeast Pdi1p, as well as *Homo sapiens* (human, Hs) HsPDI, have been mapped to the hydrophobic pocket in the b′ domain, whose substrate-binding ability seems likely to be influenced by the neighboring domains (b and a′) and by the x-linker region between b′ and a′ [40, 42–44]. The domain organization of PDIs likely contributes strongly to their substrate specificity.

**Figure 4:**
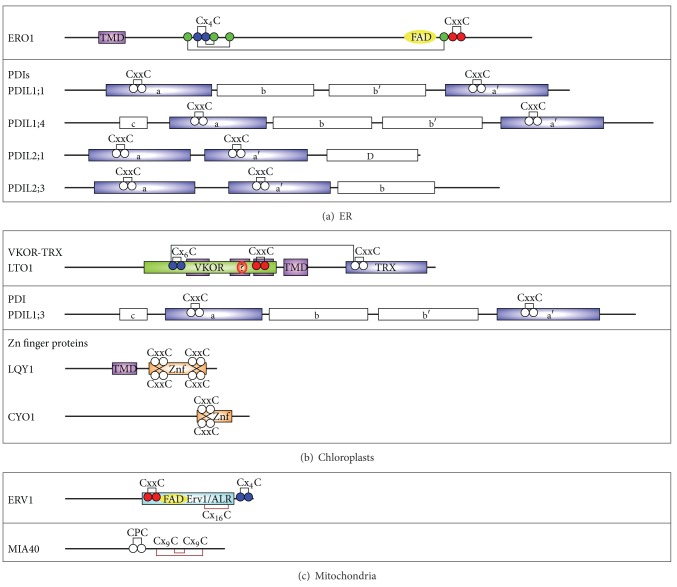
Main characterized components of the disulfide bond formation pathway in the ER, chloroplasts, and mitochondria of plant cells. (a) Schematic illustration of ERO1 and PDIs in the plant ER. (b) Schematic illustration of a VKOR homolog (LTO1) and thiol-disulfide oxidoreductases (PDIL1;3, LQY1, and CYO1) in chloroplasts. (c) Schematic illustration of ERV1 and MIA40 in mitochondria. In disulfide-generating enzymes (ERO1, LTO1, and ERV1), the predicted active-site Cys pairs, shuttle Cys pairs, and regulatory Cys pairs are indicated with red, blue, and green circles, respectively. The FAD cofactor is shown in yellow. No cofactor of LTO1 has been identified yet (question mark circled in orange). In disulfide carrier proteins and TRX domain of LTO1, the predicted redox-active Cys pairs (CxxC in TRX domains and Zn finger domains, CPC in MIA40) are indicated with white circles. The predicted structural Cys pairs in MIA40 (Cx_9_C) and ERV1 (Cx_16_C) are indicated with brown lines. Redox-active TRX (a and a′ domains; blue boxes), redox-inactive TRX (b and b′; white boxes), *α*-helical D (white box), VKOR (green box), DnaJ Zn finger (Znf; orange boxes), and Erv1/ALR (cyan box) domains are predicted by NCBI Conserved Domain searches (http://www.ncbi.nlm.nih.gov/Structure/cdd/cdd.shtml). Transmembrane helices (TMD; purple boxes) are predicted by TMHMM v. 2.0 (http://www.cbs.dtu.dk/services/TMHMM/). Acidic N-terminal c domains are also indicated (white boxes). Amino acid sequence data can be found in the GenBank/EMBL databases under the following accession numbers: OsERO1, OSJNEc05N03; OsPDIL1;1, NP_001067436; AtPDIL1;4, NP_001190581; AtPDIL2;1, NP_973708; OsPDIL2;3, NP_001063331; AtPDIL1;3, NP_191056; AtLTO1, NP_567988; AtLQY1, NP_177698; AtCYO1, NP_566627; AtERV1, NP_564557; AtMIA40, NP_680211.

Whole-genome sequencing has identified 13 genes encoding PDI proteins in Arabidopsis (At), 22 in soybean (Gm), and 12 each in rice (Os) and maize (Zm) [4, 5]. Arabidopsis AtPDIL1;1 and AtPDIL1;2; rice OsPDIL1;1, OsPDIL1;2, and OsPDIL1;3; maize ZmPDIL1;1 and ZmPDIL1;2; and soybean GmPDIL-1 show the a-b-b′-a′ domain structure (Table 1; Figure 4(a)) [4, 5, 32, 45], similar to human HsPDI and HsERp57 [46]. Arabidopsis AtPDIL1;3 and AtPDIL1;4, rice OsPDIL1;4, maize ZmPDIL1;3 and ZmPDIL1;4, and soybean GmPDIL-2 show a c-a-b-b′-a′ domain structure with an acidic N-terminal domain (c domain; Table 1; Figure 4(a)) [4, 5, 45]. In contrast, PDIL2;1–2;3 members show a three-domain structure, in which two redox-active TRX domains repeated tandemly in the N-terminal region are followed by a redox-inactive domain, either a TRX-like b domain (a-a′-b domain structure; Arabidopsis AtPDIL2;2 and AtPDIL2;3, rice OsPDIL2;3, maize ZmPDIL2;3, and soybean GmPDIM [4, 5, 32, 47], similar to human HsP5 [46]) or an *α*-helical D domain, as found in human HsERp29 (a-a′-D domain structure; Arabidopsis AtPDIL2;1, rice OsPDIL2;1 and OsPDIL2;2, maize ZmPDIL2;1 and ZmPDIL2;2, and soybean GmPDIS-1 and GmPDIS-2 [4, 5, 48]) (Table 1; Figure 4(a)).

Specific members of the PDI family have been suggested to be involved in oxidative folding of storage proteins. For example, soybean GmPDIS-1 and GmPDIM (orthologs of AtPDIL2;1 and OsPDIL2;3, resp.) are localized in the ER and associate with proglycinin in the cotyledon cells [47, 48]; GmPDIL-1 and GmPDIL-2 (orthologs of AtPDIL1;1 and AtPDIL1;3, resp.), which show higher activity for refolding of reduced, denatured RNase than do GmPDIS-1, GmPDIS-2, and GmPDIM [45, 47, 48], are localized in the ER and associate with proglycinin and *β*-conglycinin [45]. Quantitative comparison of the transcript levels of maize *ZmPDIL* genes by massively parallel signature sequencing showed that *ZmPDIL1;1* is highly expressed in all organs and tissues surveyed, except for pollen [4]. In the maize endosperm, *ZmPDIL1;1* is by far the most highly expressed PDI, followed by *ZmPDIL2;3* [4]. The level of ZmPDIL1;1 is upregulated in the seeds of *floury-2* mutant, which accumulates an abnormally processed *α*-zein [49]. In *OsERO1*-knockdown mutant seeds of rice (*ero1*), which exhibit the abnormal accumulation of storage proteins and unfolded protein response-related induction of the protein-folding chaperone BiP, OsPDIL1;1 (a ZmPDIL1;1 ortholog*;* a-b-b′-a′; Figure 4(a)) accumulates to lower levels than in the wild type, whereas OsPDIL2;3 (a ZmPDIL2;3 ortholog; a-a′-b; Figure 4(a)) accumulates to higher levels than in the wild type [50].

In a recent study, we demonstrated that rice OsPDIL1;1 and OsPDIL2;3, when expressed in the same cell, show distinct subcellular localizations, substrate specificities, and functions [32]. OsPDIL1;1, whose redox activity for oxidative protein folding depends on the redox state of the catalytic active sites (Cys69–Cys72 and Cys414–Cys417), is distributed in the ER lumen of endosperm cells [32, 50] and plays an important role in the formation of disulfide bonds in the PSV-targeted storage proteins proglutelins and *α*-globulin [32, 50, 51]. OsPDIL2;3, which does not complement the function of OsPDIL1;1 in the formation of disulfide bonds in vivo, exhibits lower activity for oxidative folding of reduced, denatured *α*-globulin and RNase than does OsPDIL1;1, whereas OsPDIL2;3 exhibits higher activity for the formation of nonnative intermolecular disulfide bonds between *α*-globulin Cys79Phe mutant proteins [32]. Interestingly, the redox-active form of OsPDIL2;3 is predominantly targeted to the surface of the ER-derived PB, and this localization of OsPDIL2;3 is highly regulated by the redox state of the redox-active sites (Cys59–Cys62 and Cys195–Cys198) [32]. The knockdown of *OsPDIL2;3* inhibits the accumulation of a Cys-rich 10-kD prolamin (Figure 3) in the core of the ER-derived PB, indicating that OsPDIL2;3 has a specific function in the intermolecular disulfide bond-assisted assembly of polypeptides into the ER-derived PB [32, 52]. Note that BiP is localized on the surface of the ER-derived PB in an ATP-sensitive manner [53]. In cultured human cells, HsP5 forms a noncovalent complex with BiP and BiP client proteins [54, 55]. The high sequence similarity between human HsP5 and rice OsPDIL2;3 suggests that OsPDIL2;3 may also interact with BiP. If so, OsPDIL2;3 and BiP may be involved in recruiting a specific group of proteins to the ER-derived PB. In addition, because the knockout of *OsPDIL1;1* causes aggregation of proglutelins through nonnative intermolecular disulfide bonds and inhibits the development of the ER-derived PB and PSV [50, 51], the distinct but coordinated function of OsPDIL1;1 and OsPDIL2;3 likely supports the development of the ER-derived PB and PSV in rice endosperm.

### 2.4. Role of PDI in the Regulation of Programmed Cell Death

In vivo and in vitro evidence strongly suggests that members of the PDI family play different physiological roles in different types of plant cells. For example, Arabidopsis AtPDIL1;4 (c-a-b-b′-a′; Figure 4(a)), which restores the wild-type phenotype in the *E. coli *protein-folding mutant *dsbA*, is highly expressed in root tips and developing seeds and interacts with BiP in the ER, thereby establishing that it is involved in oxidative protein folding [56], whereas the ER-localized AtPDIL2;1 (a-a′-D; Figure 4(a)), which can restore the wild-type phenotype in a yeast *PDI1* null mutant, is highly expressed in the micropylar region of the ovule and plays a role in embryo sac development [57].

In contrast, Arabidopsis AtPDIL1;1 (PDI5; a-b-b′-a′; Figure 4(a)) may play a role in the regulation of programmed cell death (PCD), a ubiquitous physiological process that occurs in both prokaryotes and eukaryotes during development and under various stresses [58]. The PCD pathways in plants and animals share some components, including specialized Cys proteases called caspases (Cys-containing Asn-specific proteases) that function as integration points for life-or-death decisions by cells [59, 60]. Vacuoles have been proposed to play a central role in various plant PCD pathways, including developmentally controlled and pathogen-induced PCD pathways; the collapse of vacuoles releases enzymes into the cytosol that attack organelles and DNA, leading to cell death [61, 62]. Vacuole-localized VPE, which was discovered originally as a novel Cys protease responsible for the maturation of seed storage proteins [63], shares enzymatic properties with caspase-1, and both enzymes undergo self-catalytic conversion/activation from their inactive precursors (reviewed in [64]). VPE cleaves caspase-specific substrates and regulates the death of cells during the hypersensitive response and the death of limited cell layers during early embryogenesis [64, 65]. Andeme-Ondzighi et al. have shown that Arabidopsis AtPDIL1;1 acts as a redox-sensitive regulator of the activity of the noncaspase-type, PCD-related Cys proteases RD21 and CP42, which contain a redox-sensitive active site (_Gx_C_GS_C_W_) composed of two Cys residues. AtPDIL1;1, which travels from the ER via the Golgi to vacuoles (LVs and PSVs; Table 1), specifically interacts with RD21 and CP42 to prevent their premature activation during embryogenesis and to control the timing of the onset of PCD by protease activation in endothelial cells [66].

### 2.5. The ERO1 System in the Plant ER

The introduction of disulfides into substrate proteins leads to concomitant reduction of PDIs, the reduced form of which needs to be reoxidized for the next round of the reaction cycle (Figure 1). A key question is how the plant ER establishes a redox environment that favors PDI regeneration and promotes oxidative folding of nascent polypeptides. As described in the introductory section, disulfide bond formation involves the transfer of electrons from a substrate protein to a disulfide carrier protein (e.g., PDI) and then to a de novo  disulfide-generating enzyme (Figure 1) [3]. An example of disulfide-generating enzyme is the flavoenzyme Ero1p, which was first identified in yeast by mutation studies of the temperature-sensitive conditional mutant *ero1-1* [67, 68]. The ERO1 family members are highly conserved in eukaryotic organisms, including yeast, humans, and plants. The *Saccharomyces cerevisiae*,* Caenorhabditis elegans*, and *Drosophila melanogaster* genomes each encode only a single-copy *ERO1* gene. Genome duplication is observed in humans: *HsERO1*α** and *HsERO1*β** [69–71]. In most higher and lower plants, including Arabidopsis [72], maize, and moss (*Physcomitrella patens*), there are two paralogues of *ERO1* [73]. Rice contains a single ortholog of *ERO1* [50], as do *Chlamydomonas reinhardtii *and *Volvox carteri* [73].

ERO1 introduces disulfides in PDI by relaying oxidizing power from molecular oxygen to the reduced form of PDI via the ERO1 flavin cofactor [74–76]. ERO1 proteins possess two conserved catalytic Cys pairs, a shuttle Cys pair and an active-site Cys pair (Cx_4_C and CxxC, resp.; Figure 4(a)). The shuttle Cys pair of Ero1p (Cys100–Cys105; yeast Ero1p numbering) directly oxidizes the active site of Pdi1p by means of a thiol-disulfide exchange reaction, and is subsequently reoxidized by the active-site Cys pair (Cys352–Cys355; yeast Ero1p numbering) [77–79]. The active-site Cys pair of Ero1p is reoxidized by transferring their electrons to the flavin cofactor and then to molecular oxygen [74–76]. Plant ERO1 proteins share greater homology with human HsERO1*α* than with yeast Ero1p [50, 73]. Modeling analysis of Arabidopsis AtERO1 shows that the locations of the conserved Cys residues in AtERO1 are similar to the locations in HsERO1*α* (PDB ID code 3AHQ; [80]) [73]. Rice OsERO1 possesses two pairs of Cys residues, Cys134–Cys139 and Cys391–Cys394, which correspond to Cys94-Cys99 (shuttle Cys pair) and Cys394–Cys397 (active-site Cys pair) of human HsERO1*α*, respectively (Figure 4(a)) [50].

Yeast Ero1p is tightly associated with the luminal face of the ER membrane via the 127-aa C-terminal tail [67, 81]. Human HsERO1*α*, which lacks the C-terminal domain found in yeast Ero1p, contains a 23-aa signal peptide and is retained in the ER by covalent interactions with HsPDI and HsERp44 [70, 81, 82]. Although the subcellular localization of Arabidopsis AtERO1 proteins has not been characterized, AtERO1 proteins have been shown to be glycosylated [72]. Unlike ERO1 proteins from yeast and human, rice OsERO1 is localized to the ER membrane, and this localization depends on the N-terminal region (Met1-Ser55), which contains an Ala/Pro-rich sequence (Met1-Trp36) and a putative transmembrane domain (TMD; Ala37-Ser55; Figure 4(a)) [50].

We studied the functions of rice OsERO1 by means of RNAi-induced knockdown of *OsERO1* under the control of an endosperm-specific promoter. The *OsERO1*-knockdown seeds, which produce OsERO1 at dramatically reduced levels, fail to develop the typical PSV and ER-derived PB, and abnormal aggregates form instead [50]. Knockdown of *OsERO1* inhibits the formation of native disulfide bonds in proglutelins and promotes the formation of proglutelin-containing aggregates through nonnative intermolecular disulfide bonds in the ER, as does knockout of *OsPDIL1;1* [50]. These results indicate that the ERO1-dependent pathway plays an important role in the formation of native disulfide bonds in the ER of rice endosperm cells.

ERO1 proteins possess a feedback system that regulates ERO1 activity in response to fluctuations in the ER redox environment, allowing the cell to maintain the redox conditions in the ER favorable for native disulfide bond formation [83, 84]. Yeast Ero1p contains two noncatalytic, regulatory Cys pairs (Cys90–Cys349 and Cys150–Cys295); reduction of the disulfide bonds between these Cys pairs increases Ero1p activity [83]. Human HsERO1*α* possesses a different mode of regulation; HsERO1*α* activity is regulated by an internal disulfide rearrangement in which Cys94 forms a catalytic disulfide bond with Cys99 (shuttle Cys pair Cys94–Cys99) or a regulatory disulfide bond with Cys131 [80, 84]. A similar regulatory system may also exist in plant ERO1 proteins, including Arabidopsis AtERO1 and rice OsERO1, in which the Cys residues corresponding to Cys150–Cys295 of yeast Ero1p are missing but those corresponding to Cys94, Cys99, and Cys131 of human HsERO1*α* are present, as indicated by sequence similarity analysis [50].

**Figure 5:**
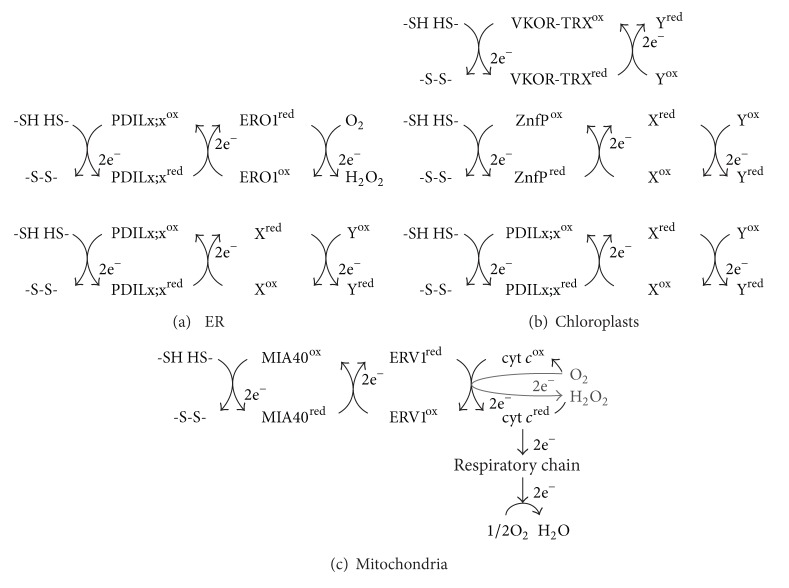
Multiple electron transfer pathways for oxidative protein folding in the ER, chloroplasts, and mitochondria of plant cells. (a) ERO1-dependent and PDI-dependent pathways in the ER. (b) VKOR-TRX-dependent, Zn finger protein-dependent, and PDI-dependent pathways in chloroplasts. LTO1 is indicated as VKOR-TRX. LQY1 and CYO1 are indicated as ZnfP. (c) ERV1-MIA40-meditaed pathway in mitochondria. One-electron carrier cytochrome *c* is indicated as cyt *c*. X^ox^ and Y^ox^ represent unidentified de novo disulfide-generating enzymes and electron acceptors, respectively. Whether de novo disulfide-generating enzymes (X^ox^) are involved in the LQY1-dependent and CYO1-dependent disulfide transfer pathways is not clear.

Additionally, the ERO1-mediated disulfide transfer system produces hydrogen peroxide, a reactive oxygen species (ROS), as a by-product (Figure 5(a)) [76]. Overexpression of ERO1 has been shown to cause a significant increase in ROS, whereas a partial reduction of ERO1 activity suppresses ROS accumulation and increases resistance to the lethal effects of ER stress [85, 86]. Although hydrogen peroxide and other ROS are cytotoxic, hydrogen peroxide sometimes plays a key role as a signal transduction messenger in eukaryotic cells [87]. High concentrations of hydrogen peroxide may cause peroxidation of membrane lipids and gradually destroy the membrane integrity in plant cells, leading to the leakage of small molecules, including water [88]. The production of hydrogen peroxide in the ER of rice endosperm cells correlates with the oxidation of sulfhydryl groups in seed storage proteins [50]. The homozygous mutant seeds of EM49, which have fewer sulfhydryl groups and generate less hydrogen peroxide than wild-type seeds, contain a higher proportion of water than wild-type seeds during seed development and show delayed seed desiccation and maturation [50]. Unlike the adjacent 2n embryo, the 3n endosperm cells, which synthesize vast amounts of disulfide-rich proteins during early seed development, are destined for PCD during seed desiccation and maturation [89]. The hydrogen peroxide generated during the formation of disulfide bonds in the ER of endosperm cells may serve as a signal for inducing PCD and subsequent seed desiccation and maturation.

Another key question is how ERO1 recognizes and oxidizes specific PDI proteins in the plant ER. Yeast Ero1p preferentially oxidizes the CxxC motif in the N-terminal TRX domain of Pdi1p [90]. In mammalian cells, specific combinations of ERO1 and PDI members drive the oxidative folding pathways. For example, human HsERO1*α* shows different affinities for human HsPDI and HsERp57 [82, 91, 92]. Crystallographic and biochemical analyses have revealed that electrostatic and hydrophobic interactions between HsERO1*α* and the PDI b′ domain allow for effective oxidation of specific PDI proteins by HsERO1*α* [80]. Although human HsERp57 shares the a-b-b′-a′ domain organization of human HsPDI, HsERp57 exhibits a different electrostatic potential on the b-b′ domain and lacks the hydrophobic pocket that is essential for substrate binding in the b′ domain [80, 93]. It remains unclear which members of the rice PDI family are specific partners of OsERO1 in the ER (Figure 5(a)). However, given that OsPDIL1;1 and OsPDIL2;3 differ in domain organization (a-b-b′-a′ and a-a′-b, resp.) and that sequence similarity between OsPDIL1;1 and OsPDIL1;4 is higher in domains a and a′ than in domains b and b′, it is plausible that OsERO1 may show different affinities for these PDI proteins.

### 2.6. Alternative Pathways for Disulfide Bond Formation in the Plant ER

The yeast *ERO1* gene is essential for viability [67, 68]. In human cells, HsERO1*α* is expressed in most cell types, whereas HsERO1*β* is expressed only in selected tissues [69–71]. Interestingly, disruption of HsERO1*α* and HsERO1*β*, which have selective and nonredundant functions in oxidative protein folding, causes only a modest delay in IgM production and is not lethal, suggesting that there are ERO1-independent pathways for disulfide bond formation in mammalian cells [94]. In fact, a candidate enzyme for a parallel ERO1-independent pathway has recently been uncovered; ER-localized peroxiredoxin (PRX) IV uses hydrogen peroxide to convert thiols into disulfides in the ER and transfers these disulfides to PDI, which in turn introduces disulfide bonds into nascent polypeptides [95]. In addition, the human glutathione peroxidase family members GPx7 and GPx8 are involved in the pathway that couples hydrogen peroxide production to disulfide bond formation; GPx7 and GPx8 are localized in the ER and preferentially oxidize PDI in the presence of hydrogen peroxide, enabling disulfide bond formation in a reduced unfolded protein [96].

Native and nonnative disulfide bonds form in *OsERO1*-knockdown mutant seeds of rice, suggesting that unidentified de novo disulfide-generating enzymes are also involved in the oxidative protein folding in the ER of endosperm cells (Figure 5(a)) [50]. In plants, PRXs play central roles in the antioxidant defense system, including thiol-based reduction of hydrogen peroxide, and in the dithiol-disulfide redox regulatory network [97]. The members of the plant PRX family are localized in chloroplasts, mitochondria, the cytosol, and the nucleus, but no ER-localized PRX has yet been identified in plants [97–99]. Among the putative TRX-dependent peroxidases of plants, glutathione-dependent peroxidase Gpx5 has been predicted to show dual localization to the ER and cytosol [98].

## 3. Disulfide Bond Formation in Chloroplasts

### 3.1. Role of PDI in Chloroplasts

Because it is derived from a cyanobacterial ancestor, the chloroplast combines prokaryotic and eukaryotic features of gene expression. Thousands of chloroplast proteins, including photosynthesis-related proteins, are encoded by nuclear genes and are synthesized in the cytoplasm. Cytoplasmically synthesized chloroplastic precursors must be unfolded for translocation across the chloroplastic double envelope membranes [100]. Most of the unfolding takes place within the membranes themselves, and proteins are then refolded before localizing to their target destinations. In addition to the nuclear-encoded chloroplast proteins, there are also approximately 100 chloroplast proteins encoded by the organelle's own genome [101]. The transcription of chloroplast genes is driven by plastid transcription systems, nuclear-encoded and plastid-encoded plastid RNA polymerases [102].

Oxygen-evolving photosynthetic organisms are subject to irreversible light-induced damage. Photodamage occurs primarily in PSII in the thylakoid membranes and leads to inactivation of photosynthetic electron transport and subsequent oxidative damage of the reaction center proteins [103]. To maintain PSII activity, the damaged D1 and D2 proteins in the PSII reaction center are degraded and replaced, and expression of plastid-encoded *psbA* and *psbD* (which encode D1 and D2, resp.) is rapidly activated at the level of transcription in response to light (reviewed in [102, 104]). In chloroplasts, reducing equivalents are generated from light by PSII and PSI and are transferred to the ferredoxin-TRX system, which in turn reduces regulatory redox-active sites of target proteins, including key proteins involved in carbon dioxide assimilation and ATP synthesis [13, 105]. It has been suggested that a reductive signal transduced by TRX activates a 5′ protein complex with high affinity for the 5′ UTR of *psbA* mRNA [14]. The genomes of the green algae *Chlamydomonas reinhardtii* and *Volvox carteri *contain 8 and 7 genes encoding PDI proteins, respectively [5]. *Chlamydomonas reinhardtii* RB60, which contains two CGHC motifs and the C-terminal KDEL signal, functions as a redox-responsive translational regulator in chloroplasts [106, 107]. The reduction-oxidation of RB60, located in a complex bound to the 5′ UTR of *psbA* mRNA, plays a key role in determining the rate of *psbA* translation [106, 108].

Arabidopsis AtPDIL1;3 (Figure 4(b)), which shows high sequence similarity to RB60 and contains the KDEL signal but not a cleavable chloroplast-targeting signal, is localized to the stromal-starch interface in chloroplasts, in addition to the ER [109–111]. The expression of *AtPDIL1;3 *is upregulated by light during chloroplast development, and this upregulation is correlated with starch synthesis [109, 112]. AtPDIL1;3 likely plays an important role in protein folding or in enzyme regulation of proteins associated with starch metabolism in chloroplasts [109].

### 3.2. VKOR Homolog-Dependent Disulfide Transfer Pathway in Chloroplasts: Roles in Assembly of the Photosystem Complexes

Although plants are subject to irreversible photodamage, as described above, they have evolved a highly specialized repair mechanism that restores the functional status of PSII. PSII repair requires disassembly and reassembly of a thylakoid membrane protein complex composed of dozens of proteins (including the reaction center proteins and oxygen-evolving proteins of PSII) and hundreds of cofactors and the breakage and reformation of disulfide bonds among PSII proteins [15, 113, 114]. Recently, a disulfide-generating VKOR homolog has been demonstrated to play important roles in assembly of the photosystem complexes in chloroplasts.

In mammals, VKOR catalyzes a step in the vitamin K cycle, which occurs in the ER membrane and is required for sustaining blood coagulation. Human HsVKOR, an integral membrane protein of the ER, consists of four TMDs with the same membrane topology as the core of a bacterial homolog of VKOR from *Synechococcus* sp. [115, 116]. The physiological redox partner of HsVKOR is a membrane-anchored member of the PDI family, with which HsERO1*α* interacts only poorly [116]. VKOR is a member of a large family of homologs that are present in various organisms, including vertebrates, plants, bacteria, and archaea [117]. All homologs contain an active-site CxxC motif and an additional pair of loop Cys residues [115, 117]. In some homologs from plants and bacteria, including Arabidopsis, cyanobacteria *Synechococcus* sp., and *Synechocystis* 6803, the domain homologous to HsVKOR is fused with the TRX-like domain typical of the PDI family (Figure 4(b)) [115, 117–119]. A crystal structure of the *Synechococcus* VKOR has revealed that electrons are transferred from a substrate protein to the reduced Cys pair (Cys209–Cys212) of the soluble C-terminal TRX-like domain and then to the two conserved loop Cys residues (Cys50 and Cys56) of the VKOR domain. Subsequently, electrons are transferred from the loop Cys pair (Cys50–Cys56) to the active-site CxxC pair (Cys130–Cys133) [115]. Like *E. coli *DsbB, VKOR accepts the oxidizing power from quinone. The C-terminal Cys residue (Cys133) in the active-site Cys pair is located on the side of the ring of quinone [115].

Arabidopsis VKOR homolog (designated LTO1) is a thylakoid membrane-localized protein that is composed of one VKOR domain, one TRX domain, and four TMDs (Figure 4(b)), as found in the *Synechococcus* VKOR, and catalyzes the formation of disulfide bonds (Figure 5(b)) [119–121]. LTO1 is required for the assembly of PSII by means of the formation of a disulfide bond in PsbO, a luminal subunit of the PSII oxygen-evolving complex [120]. Additionally, LTO1 may regulate the redox state of Cys-containing proteins residing in and facing the thylakoid lumen. In vitro enzymatic assays indicate that LTO1 is active in reducing phylloquinone, a structural cofactor tightly bound to PSI [122], to phylloquinon, but does not reduce either phylloquinone epoxide or plastoquinone [119]. Whether phylloquinone is involved in the LTO1-mediated thiol-oxidation pathway in chloroplasts is not clear.

### 3.3. Zinc Finger Protein-Dependent Disulfide Transfer Pathways in Chloroplasts: Roles in Photosystem Repair and Chloroplast Biogenesis

Other chloroplast proteins for which disulfide bond-forming activity has been reported are zinc finger-like proteins LQY1 [123] and CYO1 [124] (Figures 4(b) and 5(b)). The LQY1 protein, which is localized in the thylakoid membranes and binds to the PSII core monomer, is suggested to be involved in maintaining PSII activity and regulating the repair and reassembly of PSII under high-light conditions [123]. Arabidopsis mutants that lack the LQY1 protein show elevated accumulation of ROS and reduced levels of PSII-light-harvesting complex II supercomplex under high-light conditions [123]. Proteins homologous to Arabidopsis CYO1 are widely found in plants, including rice, maize, and soybean, but not in moss and algae [124]. The levels of the *CYO1* transcript and the CYO1 protein increase in response to light, and the CYO1 protein is localized to the thylakoid membranes in Arabidopsis [124]. The *cyo1* mutation does not affect the biogenesis of etioplasts under dark conditions but severely impairs the development of thylakoid membranes under light conditions, indicating that CYO1 plays an important role in chloroplast biogenesis [124]. Taken together with the evidence that LQY1 and CYO1 each contain a zinc finger motif (Figure 4(b)) similar to that in *E. coli *DnaJ, bind Zn^2+^, and exhibit PDI-like activity [123–125], the above data suggest that the PDI-like activity (including disulfide isomerization activity and chaperone activity) of LQY1 and CYO1 may be required for the repair of PSII and thylakoid biogenesis, respectively.

## 4. Disulfide Bond Formation in Plant Mitochondria

In mitochondria, which are also surrounded by a double membrane, the disulfide transfer pathway for oxidative protein folding is linked to the redox-regulated import of precursor proteins. Mitochondria play critical roles in numerous cellular processes, including energy metabolism and PCD. Mitochondria are derived from bacterial endosymbionts, and their genomes possess limited coding capacity (approximately 40–50 genes) [126]; thus, they largely depend on the energy-dependent import of nuclear-encoded proteins that are synthesized in the cytosol. The translocase of the outer membrane, designated the TOM complex, is the main entry gate for importing mitochondrial preproteins. In yeast, N-terminal presequence-carrying and hydrophobic precursor proteins are initially recognized by Tom20 and Tom70, respectively; subsequently transferred to the central receptor Tom22; and then inserted into the channel of the central component Tom40 [127]. Among the four subsequent sorting pathways leading to intramitochondrial destinations, the mitochondrial intermembrane space (IMS) import and assembly machinery (MIA) directs small, Cys-rich precursors into the IMS [127]. The MIA pathway is a redox-driven import pathway that requires the cooperative function of the essential IMS proteins. Yeast Mia40 binds to the precursors of IMS proteins with either twin Cx_3_C or Cx_9_C motifs, including members of the inner mitochondrial membrane chaperone complex (TIM) Tim8–Tim13. In yeast, a ternary complex composed of Mia40 (disulfide carrier protein), the FAD-bound sulfhydryl oxidase Erv1 (disulfide-generating enzyme), and the substrate facilitates the relay of disulfides from Erv1 to the precursor protein via Mia40; and the reduced form of Mia40 is reoxidized by Erv1, which transfers electrons via cytochrome *c* (one-electron carrier) to the respiratory chain and then to molecular oxygen or can use molecular oxygen directly, resulting in the generation of hydrogen peroxide (Figure 5(c)) [127–130].

Plant orthologs of yeast Mia40 and Erv1 have been found: Arabidopsis AtMIA40 and AtERV1 each contain a highly conserved redox-active Cys pair (CPC motif in AtMIA40, CxxC in AtERV1; Figure 4(c)) and structural Cys pairs (two Cx_9_C in AtMIA40, Cx_16_C in AtERV1; Figure 4(c)) and localize to mitochondria [131, 132]. Arabidopsis AtERV1 also contains noncovalently bound FAD (Figure 4(c)) and shows sulfhydryl oxidase activity [132]. Conservation of Mia40 and Erv1 orthologs and the presence of MIA import pathway substrates in yeast, animals, and plants suggest the common use of the MIA pathway [133]. Both Mia40 and Erv1 are essential in *Saccharomyces cerevisiae* [134, 135], and deletion of Arabidopsis AtERV1 causes embryonic lethality [131]. However, deletion of AtMIA40 in Arabidopsis causes no clear phenotype [131]. Additionally, unlike yeast Mia40, Arabidopsis AtMIA40, which is localized to both mitochondria and peroxisomes, does not seem to play an essential role in the import and/or assembly of small Tim proteins in mitochondria. Instead, the loss of AtMIA40 leads to the absence from mitochondria of two IMS-localized proteins, copper/zinc superoxide dismutase (CSD1; which has a dissimilar Cys pair from the Cys motif found in Mia40 substrates) and the chaperone that inserts copper into CSD1, although both are still successfully imported into mitochondria [131]. Loss of AtMIA40 also results in a decrease in the capacity of complex I and the efficiency of the assembly of subunits into complex I [131]. Arabidopsis AtERV1, which is unable to substitute for the yeast enzyme in vivo, contains both the common features of the Erv1/ALR protein family and unique features. AtERV1 lacks CxxC (shuttle Cys pair) in the N-terminal domain found in yeast Erv1 and human ALR, but contains a C-terminal Cx_4_C motif conserved among plant ERV1 homologs, which is likely to function in the interdisulfide relay [132]. MIA40 and ERV1 may thus play both conserved and distinct roles in plants.

## 5. Disulfide Bond Formation Outside the Cell

Quiescin sulfhydryl oxidase (QSOX) is an atypical disulfide catalyst, localized at various subcellular locations or outside the cell. Mammalian QSOX proteins are localized to the Golgi in the post-ER secretory pathways or secreted from cells [136–138], and Arabidopsis AtQSOX1, when expressed in the leaf epidermis, is found in the cell wall rather than the plasma membrane (Table 1) [139]. QSOX, in which the Erv domain has been linked with a redox-active TRX domain during evolution, catalyzes both de novo disulfide generation and disulfide transfer. Generation and transfer of disulfide bonds by QSOX are mediated by two redox-active Cys pairs, one in an Erv domain and the other in a TRX-like domain. Intramolecular disulfides are catalytically generated at the noncovalently bound FAD-proximal Cys pair in the Erv domain of QSOX and are in turn transferred to the Cys pair in the TRX domain by means of interdomain electron transfer, which is followed by intermolecular thiol-disulfide exchange between QSOX and a substrate [140–142]. AtQSOX1 contains a CxxC motif in the TRX domain, and this motif exhibits sulfhydryl oxidase activity on the small-molecule substrate dithiothreitol but not electron transfer activity from the TRX domain to the Erv domain [143]. The expression of *AtQSOX1* in leaves is upregulated by K^+^ starvation, and AtQSOX1 plays a role in regulating cation homeostasis by activating root systems that load K^+^ into the xylem [139]. This K^+^ efflux system may be regulated by AtQSOX1-mediated oxidation of sulfhydryl groups of the transporter at the external side of the plasma membrane [139].

In conclusion, plant cells have evolved multiple and distinct oxidative protein-folding systems in the ER, chloroplasts, and mitochondria. These systems, which require the cooperative functions of disulfide-generating enzymes and disulfide carrier proteins, support cell function and development and protect against environmental fluctuations. Individual disulfide carrier proteins (e.g., PDIs) recognize specific sets of substrate proteins. Current interest lies in uncovering the broad and specific network for oxidative protein folding in plant cells. Further study is needed to define the functions of known disulfide-generating enzymes that supply oxidizing power to specific sets of disulfide carrier proteins (e.g., PDIs), to discover additional such enzymes, and to identify the final electron acceptors in the oxidative protein-folding systems in plant cells.

## Figures and Tables

**Table 1:** Components of the system for disulfide bond formation in higher plants.

Species	Name	Domain	Active sites	Location
Disulfide-generating enzymes				
Rice	ERO1		C_x4_C, C_xx_C	ER^m^
Arabidopsis	ERV1	Erv1/ALR	C_xx_C, C_x4_C	M
Arabidopsis	LTO1	VKOR, TRX	C_x6_C, C_xx_C, C_xx_C	T^m^
Arabidopsis	QSOX1	TRX, Erv1/ALR	C_xx_C, C_xx_C, C_xx_C	W

Thiol-disulfide oxidoreductases				
PDI family proteins				
Rice	OsPDIL1;1	a-b-b′-a′	C_GH_C, C_GH_C	ER^L^
Rice	OsPDIL2;3	a-a′-b	C_GH_C, C_GH_C	PB^ER^
Arabidopsis	AtPDIL1;1	a-b-b′-a′	C_GH_C, C_GH_C	ER, G, LV, PSV
Arabidopsis	AtPDIL1;3	c-a-b-b′-a′	C_GA_C, C_GH_C	C, ER
Arabidopsis	AtPDIL1;4	c-a-b-b′-a′	C_GH_C, C_GH_C	ER, G, N, V, W
Arabidopsis	AtPDIL2;1	a-a′-D	C_GH_C, C_GH_C	ER^L^
Soybean	GmPDIL-1	a-b-b′-a′	C_GH_C, C_GH_C	ER^L^
Soybean	GmPDIL-2	c-a-b-b′-a′	C_GH_C, C_GH_C	ER^L^
Soybean	GmPDIS-1	a-a′-D	C_GH_C, C_GH_C	ER^L^
Soybean	GmPDIS-2	a-a′-D	C_GH_C, C_GH_C	ER^L^
Soybean	GmPDIM	a-a′-b	C_GH_C, C_GH_C	ER^L^
Zn finger proteins				
Arabidopsi*s *	LQY1	DnaJ Zn finger	(C_xx_C_xGxG_)_4_	T^m^
Arabidopsis	CYO1	DnaJ Zn finger	(C_xx_C_xGxG_)_2_	T^m^
Disulfide carrier protein				
Arabidopsis	MIA40		C_P_C	M

Disulfide-generating enzymes and disulfide carrier proteins from rice (*Oryza sativa*), Arabidopsis (*Arabidopsis thaliana*), and soybean (*Glycine max*) characterized to date. C, chloroplasts; ER, endoplasmic reticulum; ER^L^, ER lumen; ER^m^, ER membranes; G, Golgi; LV, lytic vacuole; N, nucleus; M, mitochondria; PB^ER^, ER-derived protein body; PSV, protein storage vacuole; T^m^, thylakoid membranes; V, vacuole; W, cell wall.

## Abbreviations

ER: Endoplasmic reticulum
ERO1: ER oxidoreductase 1
PB: Protein body
PCD: Programmed cell death
PDI: Protein disulfide isomerase
PRX: Peroxiredoxin
PSV: Protein storage vacuole
QSOX: Quiescin sulfhydryl oxidase
ROS: Reactive oxygen species
TMD: Transmembrane domain
TRX: Thioredoxin
VKOR: Vitamin K epoxide reductase
VPE: Vacuolar processing enzyme.
